# Parental Perceptions Toward Using Online Video Consultations With Pediatricians: Insights, Barriers, and Pathways to Equitable Adoption

**DOI:** 10.1155/ijta/8874943

**Published:** 2025-09-19

**Authors:** Osnat Bashkin, Tamar Shalom, Eden-Kim Admon, Rinat Rosenblum, Yoram Blachar, Tal Oron-Gilad

**Affiliations:** ^1^Department of Public Health, Ashkelon Academic College, Ashkelon, Israel; ^2^Department of Health Systems Management, The College of Law and Business, Ramat Gan, Israel; ^3^Department of Industrial Engineering and Management, Ben-Gurion University of the Negev, Be'er-Sheva, Israel; ^4^Division of Community Medical Services, Clalit Health Services, Tel Aviv, Israel

**Keywords:** children, parents, pediatric care, telemedicine, video consultation

## Abstract

**Introduction:** Online video consultation (VC) use in pediatric care has increased recently, particularly following the COVID-19 pandemic. However, understanding parental perspectives on VC use with pediatricians, especially across geographical regions, remains crucial for effective VC implementation and reducing healthcare disparities.

**Methods:** This mixed methods study combined an online survey (*n* = 96) and two focus groups (*n* = 17) to examine parental perceptions of VC with pediatricians in Israel. Based on the Telehealth Usability Questionnaire, the survey assessed user experience, usefulness, and satisfaction. Focus groups explored themes related to children's experiences, remote diagnosis, patient–physician communication, and VC use during emergencies.

**Results:** While 82% of parents believed that VC saved time and 67% felt it improved access to healthcare, only 50% thought it met their healthcare needs. Parents in peripheral regions rated the usefulness of VC higher than those in central areas, particularly during times of security concerns. However, central region parents reported better user experiences. Parents with prior VC experience showed higher satisfaction and future use intention than non-preusers. Focus groups revealed that children often felt more comfortable with VC due to the familiar home environment, although parents expressed concerns about remote diagnosis accuracy and physician communication quality.

**Discussion:** Our findings highlight geographic disparities in VC adoption and emphasize the need for targeted interventions to improve technical support and infrastructure in peripheral regions. While VC shows promise for enhancing healthcare accessibility, particularly during emergencies, success requires addressing technical barriers, strengthening privacy measures, and establishing clear guidelines for remote pediatric care delivery.

## 1. Introduction

Telepediatric health services are aimed at covering the various stages of pediatric care without requiring physical meetings, enabling healthcare services to children at a distance to overcome geographical and logistical challenges [1]. Additionally, telepediatrics allows accessible and continuous patient monitoring, guidance, and direction. This subspecialty of telemedicine enables support and equal treatment for patients from different populations, making medical services more accessible [2]. Furthermore, there is a growing need for pediatric healthcare telemedicine as a result of challenges resulting from the COVID-19 pandemic, limited pediatric healthcare accessibility in rural areas, challenges in children's chronic illness management, the lack of pediatric specialists, and difficulties associated with traveling with children [3, 4].

Video consultation (VC) is a medical consultation conducted via internet-based video calls between the treating physician and the patient that enables remote examination, virtual home visits, and the involvement of family members and caregivers. VC is a telemedicine solution to reduce physicians' workload and prevent availability issues [5]. A study of medical visits using VC found that approximately 60% of the visits were rated as good as or better than face-to-face visits. The researchers found that VC was suitable for delivering laboratory results, medication adjustment, and administrative tasks such as providing patients with medical documents and monitoring chronic diseases, including mental healthcare [6]. VC can support healthcare delivery to remote areas. A study investigating VC adoption in primary care in rural areas showed benefits for remote rural populations facing general practitioner shortages, time and travel savings, flexibility in integrating medical consultations into patients' daily routines, prevention of exposure to various infections, and increased focus on the patient during consultations [5].

The literature shows evidence of satisfaction among physicians using VC. A systematic review found that more than 88% of over 700 family physicians felt that VC enabled successful clinical decision-making. Furthermore, 50% of the family physicians believed that VC was as good as or better than face-to-face consultation, primarily due to distancing during the COVID-19 period. About 41% of the family physicians preferred face-to-face consultation [7]. Another advantage for physicians is the ability to observe patients in their natural environment and in the presence of family members who may be essential to the meeting [2]. However, other studies mention physicians' concerns regarding the implications of VC. A study conducted in Israel examining perceptions and difficulties in telemedicine use by pediatricians noted challenges related to remote diagnosis, the initial treatment of patients without prior acquaintance, technical difficulties, and a moral conflict between meeting parents' expectations in providing remote care while maintaining treatment standards, which may not necessarily be achieved through VC [8]. The researchers indicated that the most suitable medical treatments for VC use were relatively simple medical issues and those requiring short treatments.

Among patients, opinions are divided. In a qualitative study, patients noted efficiency and convenience, including reduced travel times, the ability to attend medical consultations without taking time off work, the absence of waiting room delays, and improved access to various specialists. Additionally, patients appreciated the ecological sustainability of such meetings and perceived this service as being reliable and safe. Patients also reported experiencing less discomfort and pressure in VC meetings than in face-to-face meetings [2]. However, several studies raise patient concerns: (1) about personal medical data being more vulnerable to breaches and disclosure by others [5] and (2) about the need for personal relationships and direct interaction, assuming that physicians may provide better care through personal contact [9]. Some parents reported that, in situations involving psychological stress around a particular issue, there may be a preference for personal face-to-face treatment [10]. Moreover, VC requires technological access, so technologically challenged people may have trouble using this platform [5, 11, 12]. A systematic review noted that patients complain about decreased personal interaction in VC meetings compared to physical clinic visits. A preference for face-to-face consultations over VC was observed, particularly among patients with chronic disease and older ages [7]. Tully et al. [13] examined barriers and facilitating factors for implementing telepediatrics and showed that parents preferred remote consultation only with their regular physician. The importance of treatment continuity with the same physician varied among users and depended on the complexity of the health issue. Furthermore, parents who tended to use telemedicine services were those with previous telemedicine consultation experience or higher openness to telemedicine consultation. The main barrier identified was the technological reliability and inadequacy of the VC tool for various types of examinations from a logistical perspective.

Pediatric care presents unique challenges and considerations in telemedicine implementation, as it involves not only the direct healthcare provider–patient interaction but also the crucial role of parents as mediators in this process. While the COVID-19 pandemic accelerated the adoption of telemedicine services, there remains a critical need to understand whether this digital transformation effectively meets the needs of pediatric care, where physical examination has traditionally been considered essential. Understanding parental perspectives across different geographical areas is crucial to inform policy decisions about resource allocation, technology implementation, and healthcare delivery models that could effectively reduce healthcare disparities between central and peripheral regions.

In the current study, we examined parental perspectives regarding VC use with pediatricians, employing a mixed methods approach combining a survey and focus groups. This research is aimed at identifying factors influencing the parental adoption of VC services and understanding barriers and facilitators to VC implementation in pediatric care. Additionally, we sought to examine whether geographical location impacts parents' willingness to use VC services, focusing on whether those living in peripheral regions, with limited access to pediatric specialists, differed from those living in central regions.

## 2. Methods

### 2.1. Design

This study comprised an online survey and two focus groups with parents.

### 2.2. Settings and Participants

A survey examining parents' perceptions and experiences of VC with pediatricians was distributed in April 2024. A secure link and a brief description of the project were disseminated on social media networks, and individuals were invited to complete the survey. For the survey component, a minimum sample size of 85 participants was calculated for detecting medium effect sizes (Cohen's *d* = 0.5) with 80% power and *α* = 0.05 for comparing two independent groups (peripheral vs. central regions). Focus group participants were recruited via convenience sampling using advertising posts. The inclusion criterion was being a parent of a minor. The focus group meetings took place between May and June 2024 and were conducted in accordance with COREQ guidelines [14]. Seventeen parents participated in the focus group discussions. Ten parents who participated in the focus group discussion lived in Ramat Gan, a city in the center of Israel (eight women and two men; age range, 33–41 years), and seven parents who participated in the focus group discussion lived in Be'er-Sheva, a peripheral southern city (seven women; age range, 33–47 years). The sample size for focus groups (*n* = 17 across two groups) was determined based on established qualitative research principles. Guest et al. [15] suggest that thematic saturation typically occurs within 6–12 interviews for homogeneous populations. The geographic stratification (central vs. peripheral regions) ensured representation of diverse perspectives while maintaining manageable group sizes for meaningful discussion. Data saturation was assessed during analysis.

### 2.3. Data Collection Tools

#### 2.3.1. Survey

The survey was developed using Qualtrics XM and was based on the Telehealth Usability Questionnaire (TUQ) [16] adapted for the pediatric context by modifying language to reflect the parents' perspective and contextualizing items for pediatric care scenarios. Three items were excluded as not applicable, resulting in an 18-item instrument. The adapted survey underwent content validation by three experts (pediatrician, health systems researcher, and human factors engineer) and pilot testing with 10 parents before distribution to assess comprehensibility and identify any technical issues. Minor clarifications were made to the question wording based on pilot feedback. The final modified survey included multiple-choice questions and 5-point Likert scales, ranging from 1 (*strongly disagree*) to 5 (*strongly agree*) (see Supporting Information 1: File S1—survey). Topics of interest included participants' demographics, VC accessibility, frequency of VC use with the pediatrician, user experience during the interaction, usefulness, general satisfaction, and the likelihood of using it again or recommending it to other parents. Parents who reported no experience referred to the expected usefulness and satisfaction and did not answer questions related to user experience and interaction with the tool. The survey items were divided into three dependent variables, such that each variable was calculated by the means of the items comprising the variable:

1. User experience and usability during VC with the personal pediatrician: Items 1–8 and Item 14 (*α* = 0.67)
2. Usefulness or expected usefulness of the VC tool with the personal pediatrician: Items 9–13 (*α* = 0.85)
3. Satisfaction or expected satisfaction and intention to use VC with the personal pediatrician in the future: Items 15–18 (*α* = 0.77).

#### 2.3.2. Focus Groups

Two researchers (E.K. and R.R.) were trained to conduct focus groups and facilitated each focus group session. The session was semistructured and lasted about 1 h, with a predefined list of open-ended questions being asked during each session (see Supporting Information 2: File S2—focus group guide). To ensure trustworthiness, the same focus group discussion guide was used in both sessions; this guide was developed with a pediatrician (YB) and was based on the main survey findings. The sessions were audio-recorded upon receiving the consent of each participant. The audio recordings were then transcribed verbatim and deidentified.

### 2.4. Ethics

The study received approval from the Ben-Gurion University Ethics Committee (11/2024). No personally identifiable data was collected for research purposes, and participants could withdraw from the study at any time. Eligible participants gave their informed consent for inclusion in the study and were informed about anonymity, data protection, and privacy. The study researchers' contact information was provided. There was no incentive to participate.

### 2.5. Analysis

Data analysis was carried out using R software Version 4.3.3. Reliability was examined using Cronbach's alpha. Descriptive statistics report the respondents' demographics and characteristics. Nonparametric tests were applied to analyze the significant associations between the sample characteristics and study variables (user experience and interaction, usefulness, and satisfaction). Based on Thiese et al. [17] and considering the limited sample size, a significance level of *p* < 0.10 was determined to be necessary. Qualitative free-text answers were transcribed. Descriptive data were organized into themes and subthemes, and key direct quotations were extracted. Emerging themes were discussed and reviewed by the study team during analysis.

## 3. Results

### 3.1. Survey Participants' Information

A total of 96 parents responded to the survey, of which 87.5% were women and 33.7% were living in peripheral areas of the country. Among the respondents, 46% had used VC with other physicians for general consultations, and 27% had used VC with their children's pediatrician. The respondents' characteristics are presented in Table 1.

**Figure 1:**
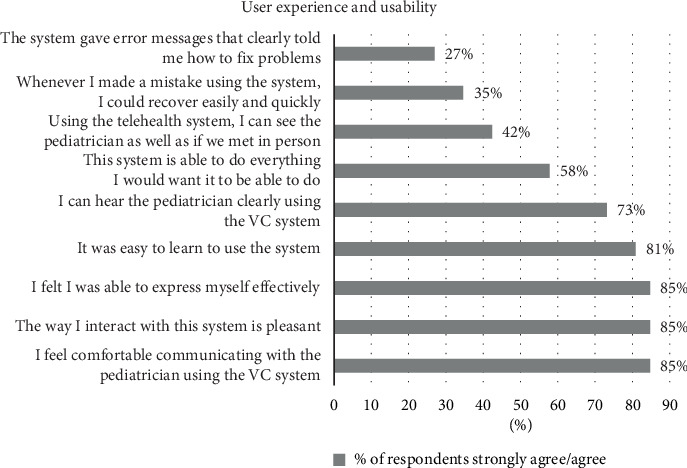
Percentages of respondents who strongly agreed/agreed with statements regarding the user experience and usability of VC.

Parents' perceptions of user experience and usability of the VC tool are presented in Figure 1. The figure illustrates the extent to which parents perceived the usability of the VC tool and their experience with it as positive.

The analysis shows that most of the respondents reported a pleasant and comfortable experience while using VC with their pediatricians. Nevertheless, parents' experience related to errors while using VC was less positive, as only 27% agreed that the system gave error messages that guided the user to fix the problems, and only 35% reported an easy and quick recovery from user mistakes.

**Figure 2:**
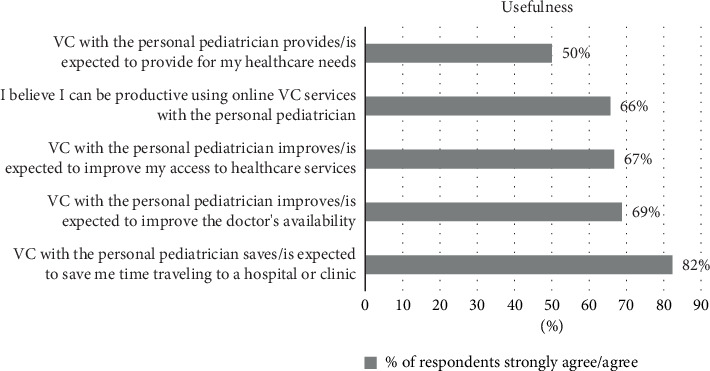
Percentages of respondents who strongly agreed/agreed with statements regarding the usefulness of VC.

Figure 2 illustrates the extent to which parents perceive the use of the VC tool with their pediatrician as being useful. The figure presents the percentages of respondents who either strongly agreed or agreed with a series of statements related to the usefulness of the VC tool.

The analysis shows that, while most of the respondents believe that using VC with their pediatrician may save time (82%) and may improve access to healthcare services (67%), only half of the respondents think that VC can meet their healthcare needs.

**Figure 3:**
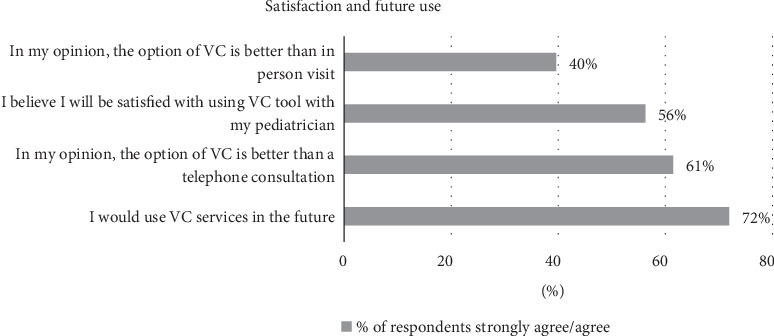
Percentages of respondents who strongly agreed/agreed with statements regarding user satisfaction and future use of VC.

Figure 3 illustrates the extent to which parents rate their satisfaction and intention to use VC in the future.

As can be seen in Figure 3, while most of the respondents mentioned that they would use the VC services in the future (72%) and 56% were satisfied with the tool, just 40% believed that VC is better than in-person visits in the pediatrician clinic.

Nonparametric tests were conducted to examine differences in the survey respondents' perceptions and experiences. The average rates of respondents' user experience, usefulness, and satisfaction are presented in Table 2.

As shown in Table 2, significant differences were found between parents who had an academic degree and parents who did not in terms of both usefulness and satisfaction. Parents who had an academic degree rated the usefulness of VC lower than parents who did not have a degree (3.8 vs. 4.1, *p* < 0.1). They also reported lower satisfaction and intention to use VC in the future (3.5 vs. 3.9, *p* < 0.1). In addition, parents who previously used only telephone calls or online chat with any other physician rated the experience and usability of the VC tool higher than parents who previously used VC with any other physician (4.1 vs. 3.6, *p* < 0.1). However, parents who previously used only telephone calls or online chat with any other physician rated the usefulness of the VC tool as lower (3.8 vs. 4.2, *p* < 0.1) and reported lower satisfaction than parents who previously used VC with any other physician (3.5 vs. 3.9, *p* < 0.1).

### 3.2. Focus Group Findings

Four main themes emerged from the thematic analysis: (1) the child's experience with VC versus in-clinic meetings with the pediatrician, (2) the remote diagnosis, (3) patient–pediatrician communication during VC, and (4) VC use during emergencies or unstable circumstances.

#### 3.2.1. The Child's Experience With VC Versus In-Clinic Meetings With the Pediatrician

Parents shared that their children enjoy visiting the clinic. For example, one woman (Participant 8), a mother living in central Israel, stated: “My daughter really loves going to the clinic. There are games she plays while waiting, she always gets a surprise at the end of the visit, and the whole experience is exciting for her.”

However, some parents argued that their children feel more secure and comfortable with remote VC with the pediatrician, primarily because of the familiar home environment. Participants mentioned that the proximity to parents and distance from medical equipment, which can cause anxiety among children, can create a greater sense of comfort in children. Participant 4 from southern Israel added that VC with the pediatrician is less threatening: “I think they're less afraid from a distance when they do not have to lay down on the treatment bed at the clinic.”

Participants claimed that, although clinic visits can be enjoyable due to the attention and quality time with parents and positive interaction with the physician, children may prefer remote medical consultations via video as they provide distance and protection from a “threatening” medical environment, as well as security due to the familiar environment and comfort of home.

#### 3.2.2. The Remote Diagnosis

Participants expressed varying opinions regarding the reliability of remote diagnosis via VC and the comfort and usability of the technology. Some participants noted that remote diagnosis is helpful, especially in non–life-threatening situations, and reduces the need to visit the clinic. For example, Participant 1, a mother from the periphery, stated: “In a non-life-threatening situation, I would try to get some online opinion before dedicating my entire workday to traveling to the clinic. It also helps with the long waiting time at the clinic. If it's our regular pediatricians, I trust them.”

However, other participants argue that remote diagnosis is not always accurate and reliable, especially for young children or situations requiring physical examination: “I would be much more relaxed if the doctor examined the child in person. I think it's also related to the child's age; the younger they are, the more they need the doctor physically at the clinic” (Participant 3, a mother living in the southern periphery). These findings show the need for clear guidelines on when remote diagnosis is appropriate.

#### 3.2.3. Patient–Pediatrician Communication During VC

Most of the two focus group participants feared that remote medical consultations might cause physicians to be less focused and sometimes rush to end the meeting. Participant 3, from the center of the country, noted: “I feel that the doctor is somewhat less patient compared to meetings held at the clinic.” However, Participant 5, also from the center, is pleased with remote consultations: “It depends on the doctor. My pediatrician is amazing; I really trust her. She explains even long protocols to me and can get back to me even at midnight.”

The findings reveal the need for training healthcare providers in remote consultation etiquette and the importance of maintaining professional standards in virtual settings.

#### 3.2.4. VC Use During Emergencies or Unstable Circumstances

Parents indicated that they fear visiting clinics for the risk of additional infections and security concerns related to situations such as war and epidemics. There was a significant difference in the level of concern about visiting clinics during wartime between parents living in the periphery versus those living in central Israel. In the southern periphery, security concerns greatly affected parents. A consensus emerged regarding concerns about clinic visits during wartime. These anxieties were particularly pronounced in locations where clinics lack protective shelters or safe rooms, as illustrated by Participant 6, a mother from Israel's southern periphery: “Our clinic has no protected space, so I prefer VC rather than visiting the clinic in such a life-threatening reality. It is much safer.”

Additionally, participants expressed significant concern about the potential exposure to infectious diseases during clinic visits, leading parents to consider remote healthcare services as an alternative to clinic attendance. As explained by Participant 1 from Israel's southern periphery: “I always say there's no need to rush to the clinic where there are infectious diseases. It's better to wait two days to see what is going on and consult via video as a temporary solution.” These findings emphasize the critical need for the expanded availability of remote VCs with pediatricians, particularly during wartime and in high-risk areas.

Furthermore, the findings reveal that anxiety about infectious disease exposure during clinic visits was prevalent among all focus group participants, with no discernible differences between peripheral and central regions. These concerns are likely to increase and promote the utilization of remote medical consultations as a means of infection prevention. Consequently, providing parents with the option to schedule video appointments appears to be essential.

The findings highlight the importance of considering geographical location in healthcare delivery and the potential for expanded telehealth services in conflict zones. There is a need for robust remote healthcare infrastructure in emergency situations and for emergency protocols integrating remote consultations.

## 4. Discussion

Our study examined parental perceptions of VC with pediatricians, focusing on user experience, usefulness, and satisfaction. Using mixed methods, our work contributes to a deeper understanding of differences in the parental perceptions of VC with pediatricians. Parental perceptions varied depending on the geographic location and distance from the clinic. In addition, differences were observed in parents' perceptions according to previous use of the VC tool, revealing both the strengths and challenges of VC adoption.

### 4.1. VC Use in Remote Areas

The findings revealed that parents viewed VC as a useful tool, particularly in terms of accessibility, convenience, and time savings, with significant differences noted between parents living in peripheral versus central regions. Parents in peripheral areas perceived greater usefulness of VC, possibly due to the increased convenience and reduced travel requirements [10, 18, 19]. However, user experience and usability were rated significantly higher by parents who lived in the center of the country and were relatively close to the pediatrician's clinic. This aligns with findings from a recent review study that showed the value of telepediatrics in rural areas [1], although Helvey et al. [20] found that rural communities in the United States showed both higher utilization and higher satisfaction with telemedicine services compared to urban areas. Similarly, studies from Australia's rural and remote health program have consistently shown that geographic isolation correlates with both higher acceptance and better user experiences with telehealth services. Our finding of infrastructure-related user experience disparities suggests that Israel's technological gaps between regions may be more pronounced than in countries with comprehensive rural telehealth investments.

Technological readiness and infrastructure may be more influential in determining user experience than the actual need for remote services [21]. Parents from peripheral regions expressed particular concern about security issues and the lack of protected spaces in clinics, leading them to view VC as a crucial safety measure rather than merely a convenience. This finding represents a novel contribution beyond traditional convenience-based models and deepens our understanding of why peripheral residents rated the usefulness of VC higher despite reporting lower user experience scores. These qualitative findings regarding unstable circumstances were particularly timely, revealing that parents viewed VC as protection from physical threats, extending pandemic-era infection control motivations [3, 4] to conflict-affected contexts, with important implications for telehealth policy in unstable regions globally. This extends beyond previous literature that primarily focused on convenience factors to show how VC can serve as a crucial healthcare delivery mechanism during times of crisis or instability.

The geographical disparity in user experience highlights the need for targeted interventions to improve the technological infrastructure and support in peripheral regions, ensuring that those who could benefit most from VC have the means to use it effectively. The potential for reducing medical anxiety among children through remote consultations shows the need for a personalized approach to healthcare delivery based on individual child preferences and the importance of offering both in-person and remote consultation options.

### 4.2. The Influence of Preuse of VC

Significant differences were also found between patients who had previously used VC with their pediatrician and those who had not yet encountered VC for pediatric care and were thus still in the process of forming behavioral intentions and attitudes toward VC in pediatric care. Parents with prior experience using VC for pediatric care perceived significantly greater usefulness of VC and reported higher satisfaction and greater intention to continue using it in the future. Previous studies support this finding, demonstrating that parents with prior experience using telehealth services tend to exhibit higher levels of acceptance and satisfaction with these tools [22, 23]. This may be due to increased familiarity with the technology and greater trust in its efficacy.

Findings among preusers of VC showed that user experience is another vital factor related to the adoption of VC in pediatrics. While parents who were preusers of VC expressed higher usefulness, satisfaction, and intention to use VC in the future, they expressed lower user experience and usability of the VC with their pediatrician. Analysis revealed that participants encountered difficulties related to technical errors and system failures. The literature indicates that technological proficiency and system stability are pivotal to user experience. Tully et al. [13] emphasized that training and technical support are key facilitators for successful telemedicine adoption. Addressing these barriers through enhanced technical support, training, and user-centered system design could significantly improve the user experience.

Our findings showed that, despite high parental satisfaction with VC, skepticism about the adequacy of VC relative to in-person visits persisted. Concerns regarding the quality of remote diagnosis and data security also emerged as potential barriers to adoption. This reflects the broader tension highlighted by Barsom et al. [24], who noted that, while patients valued the convenience of VC, some perceived it as an inferior substitute for in-person care. Several studies emphasized the need for robust privacy safeguards to ensure user trust [21, 25]. Addressing data privacy concerns through the clear communication of privacy policies and the implementation of secure, encrypted platforms could foster greater parental confidence in the system. It is important to maintain quality standards in remote healthcare and to improve technological solutions to enhance remote diagnostic capabilities.

In addition, the qualitative theme of patient–physician communication during VC revealed important insights into how the quality of virtual interactions varies significantly according to individual physician practices. While some parents reported rushed or impersonal interactions, others described highly satisfactory experiences with physicians who adapted well to the virtual format. This variability suggests that pediatrician training and standardization of VC protocols might be necessary to ensure consistent quality of care, supporting findings by Haimi et al. [8] regarding the need for clear telemedicine guidelines. Similar to previous studies that emphasized that VC is not appropriate for all patients [26–28], our findings also underscore the need for clear communication regarding the specific medical scenarios where VC is most effective. There is a need for guidelines on remote consultation duration and quality due to the potential impact on patient–physician relationships.

Unlike adult primary care studies, where Mueller et al. [5] found uniformly positive rural experiences, pediatric care presents unique challenges: parent–child–physician communication triangulation and heightened diagnostic accuracy concerns. While children felt more comfortable at home (aligning with reducing medical anxiety), parents' diagnostic concerns created a less prominent tension in adult telehealth literature [2, 7]. In addition, unlike adult studies showing linear experience–satisfaction relationships, in the pediatric contexts, parents appreciate benefits while maintaining critical awareness of limitations due to high-stakes child health decisions.

Our results suggest that ad hoc VC implementation without systematic support creates persistent usability challenges even among experienced users, supporting the need for structured onboarding programs rather than assuming experience automatically improves satisfaction.

Traditional technology acceptance models may need modification for pediatric telehealth contexts to account for (1) proxy decision-making dynamics, (2) geographic infrastructure disparities, (3) crisis-driven adoption motivations, and (4) the experience–skepticism paradox we identified.

Our results support differentiated approaches: technical support prioritization in peripheral regions and quality standardization in central areas. The geographic disparity in user experience highlights how technological solutions intended to reduce disparities can create new inequalities when infrastructure support is unevenly distributed.

### 4.3. Limitations

This study has several limitations. While sufficient for initial insights, the sample size is relatively small, which limits the generalizability of the findings to a broader population of parents in different geographic or socioeconomic contexts. To address this, we used mixed methods data collection to triangulate findings and provide a richer understanding of parental perceptions. Second, the study was conducted in a specific regional context, and cultural, technological, and healthcare system differences may affect the applicability of these findings to other countries or regions. Finally, the cross-sectional design of the study captures parental perceptions at a single point in time, making it difficult to account for how perceptions might change with increased experience or exposure to VC over time. To partially address this issue, we included participants with both prior experience and no prior experience with VC, providing a comparative perspective on potential shifts in perceptions. Future research should consider longitudinal designs to track changes in parental attitudes and explore the effects of targeted interventions aimed at improving the user experience.

## 5. Conclusions

Our findings provide valuable insights into the parental perceptions of VC with pediatricians, highlighting both the potential benefits and the barriers to adoption. Parents, particularly those in peripheral regions, view VC as a useful tool that enhances accessibility, convenience, and safety, especially in times of crisis or instability. However, the study also reveals significant disparities in user experience and technological readiness between parents in central and peripheral areas. Parents with prior VC experience demonstrated higher satisfaction, usefulness ratings, and a greater likelihood of future use, suggesting the importance of familiarization and exposure to the tool. Concerns about technical challenges, privacy, and data security remain barriers to broader adoption. These findings underscore the need for targeted interventions to improve technical support, strengthen privacy measures, and provide training for healthcare providers to ensure consistent and high-quality virtual care. Our findings also emphasize the importance of prioritizing telehealth access for geographically underserved populations to promote health equity. Policymakers and healthcare administrators should prioritize equitable access to VC, particularly for populations in underserved areas, and consider strategies to promote trust and satisfaction with digital health services. Ultimately, this study highlights the growing role of VC in pediatric healthcare and emphasizes the need for a patient-centered approach that addresses the diverse needs and expectations of parents across different geographic and socioeconomic settings.

## Figures and Tables

**Table 1:** Characteristics of the study sample (*N* = 96).

**Demographic variables**		**N** ** (%)**
Sex	Male	12 (12.5%)
	Female	84 (87.5%)

Age group, years	18–34	25 (26.0%)
	35–44	37 (38.5%)
	≥ 45	34 (35.5%)

Education	Academic degree	51 (53.1%)
	High school graduate/diploma or the equivalent	45 (46.9%)

Area of residence	Center	63 (66.3%)
	Periphery	33 (34.3%)

Number of children	1–2	48 (50.0%)
	≥ 3	48 (50.0%)

Time to reach the pediatric clinic, min	< 7	42 (43.8%)
	≥ 8	54 (56.2%)

Child's chronic disease	Yes	11 (11.4%)
	No	85 (88.6%)

Previous use of telemedicine tools with any physician	Phone call or online chat	52 (54.2%)
	Online VC	44 (45.8%)

Previous use of online VC with the personal pediatrician	Yes	26 (27.1%)
	No	70 (72.9%)

**Table 2:** Average rates of respondents' usefulness, user experience, and satisfaction.

**Demographic variables**		**User experience (** **N** = 26**)** **M (SD)**	**Usefulness (** **N** = 96**)** **M (SD)**	**Satisfaction (** **N** = 96**)** **M (SD)**
Sex	Male	3.1 (0.0)	3.9 (0.6)	3.7 (0.9)
	Female	3.8 (0.5)	4.0 (0.8)	3.7 (0.9)

Age group, years	18–34	3.6 (0.6)	3.8 (0.9)	3.7 (0.9)
	35–44	3.7 (0.6)	4.0 (0.8)	3.6 (0.9)
	≥ 45	4.0 (0.4)	3.9 (0.8)	3.8 (0.9)

Education	Academic degree	3.8 (0.4)	3.8 (0.8)^a∗^	3.5 (0.8)^a∗^
	High school graduate/diploma or the equivalent	3.7 (0.7)	4.1 (0.8)	3.9 (0.9)

Area of residence	Center	3.9 (0.4)^a∗^	3.9 (0.8)	3.7 (1.0)
	Periphery	3.5 (0.6)	4.1 (0.9)	3.7 (0.8)

Number of children	1–2	3.8 (0.5)	4.0 (0.9)	3.8 (0.9)
	≥ 3	3.6 (0.6)	3.9 (0.8)	3.6 (0.9)

Time to reach pediatrician clinic, min	< 7	4.0 (0.4)^a∗^	4.0 (0.9)	3.9 (0.9)^a∗^
	≥ 8	3.6 (0.6)	3.8 (0.8)	3.6 (0.9)

Child chronic disease	Yes	3.8 (0.1)	4.0 (0.7)	3.5 (1.0)
	No	3.7 (0.5)	3.9 (0.9)	3.7 (0.9)

Previous use of telemedicine tools with any physician	Phone call or online chat	4.1 (0.6)^a∗^	3.8 (0.9)^a∗^	3.5 (0.9)^a∗^
	Online VC	3.6 (0.5)	4.2 (0.7)	3.9 (0.8)

Previous use of online VC with the personal pediatrician	Yes	3.7 (0.5)	4.2 (0.7)^a∗^	4.0 (0.8)
	No	—	3.9 (0.9)	3.6 (0.9)

^a^Mann–Whitney test.

⁣^∗^*p* < 0.1.

## Data Availability

The data that supports the findings of this study are available from the corresponding author.

## Supporting Information

Supporting Information 1 File S1: Survey.

Supporting Information 2 File S2: Focus group guide.
