# Supplementary material for: Parental Perceptions Toward Using Online Video Consultations With Pediatricians: Insights, Barriers, and Pathways to Equitable Adoption
Source: Int J Telemed Appl. 2025 Sep 19;2025:8874943. doi: 10.1155/ijta/8874943 (PMC12473984; doi:10.1155/ijta/8874943)
Supplement: Supporting Information 1 — File S1: Survey. [file 8874943.f1.docx]

**Supplementary 1 - Survey**

1. Sex: 1. Male 2. Female
2. Age: 1. 18-34 2. 35-44 3. 45+
3. Education: 1. Academic degree 2. High school graduate, diploma or the equivalent
4. Area of residence: 1. Centre 2. Periphery
5. Number of children: 1. 1-2 2. 3+
6. Time to reach the pediatric clinic: 1. Less than 7 minutes 2. 8 minutes or above
7. Child chronic disease: 1. Yes 2. No
8. Which of the telemedicine tools have you used previously with any other physician: 1. Phone call or online chat 2. Online VC
9. Have you used before an online VC with your personal pediatrician: 1. Yes 2. No

Circle the number indicating the extent to which you agree with the following statements:

|  | Strongly disagree |  |  |  | Strongly agree |
| --- | --- | --- | --- | --- | --- |
| 1. The system gave error messages that clearly told me how to fix problems | 1 | 2 | 3 | 4 | 5 |
| 1. Whenever I made a mistake using the system, I could recover easily and quickly | 1 | 2 | 3 | 4 | 5 |
| 1. Using the telehealth system, I can see the pediatrician as well as if we meet in person | 1 | 2 | 3 | 4 | 5 |
| 1. I can hear the pediatrician clearly using the VC system | 1 | 2 | 3 | 4 | 5 |
| 1. This system is able to do everything I would want it to be able to do | 1 | 2 | 3 | 4 | 5 |
| 1. It was easy to learn to use the system | 1 | 2 | 3 | 4 | 5 |
| 1. I feel comfortable communicating with the pediatrician using the VC system |  |  |  |  |  |
| 1. The way I interact with this system is pleasant | 1 | 2 | 3 | 4 | 5 |
| 1. VC with the personal pediatrician improves/is expected to improve my access to healthcare services | 1 | 2 | 3 | 4 | 5 |
| 1. VC with the personal pediatrician improves/is expected to improve the doctor's availability | 1 | 2 | 3 | 4 | 5 |
| 1. VC with the personal pediatrician saves/is expected to save me time traveling to a hospital or clinic | 1 | 2 | 3 | 4 | 5 |
| 1. VC with the personal pediatrician provides/is expected to provide for my healthcare needs | 1 | 2 | 3 | 4 | 5 |
| 1. I believe I can be productive using online VC services with the personal pediatrician | 1 | 2 | 3 | 4 | 5 |
| 1. I felt I was able to express myself effectively | 1 | 2 | 3 | 4 | 5 |
| 1. I would use VC services in the future | 1 | 2 | 3 | 4 | 5 |
| 1. In my opinion, the option of VC is better than a telephone consultation | 1 | 2 | 3 | 4 | 5 |
| 1. In my opinion, the option of VC is better than in-person visit | 1 | 2 | 3 | 4 | 5 |
| 1. I believe I will be satisfied with using VC tool with my pediatrician | 1 | 2 | 3 | 4 | 5 |

**Thank you for your cooperation!**
