# Supplementary material for: Parental Perceptions Toward Using Online Video Consultations With Pediatricians: Insights, Barriers, and Pathways to Equitable Adoption
Source: Int J Telemed Appl. 2025 Sep 19;2025:8874943. doi: 10.1155/ijta/8874943 (PMC12473984; doi:10.1155/ijta/8874943)
Supplement: Supporting Information 2 — File S2: Focus group guide. [file 8874943.f2.docx]

**Supplementary 2 - Focus groups guide:**

1. Do you think there's a difference between having an appointment at the clinic versus via video? What's the difference?
2. In which situations would you choose to go to the clinic instead of video? Why? When is it advisable to use video and when not? What considerations should a parent take into account?
3. Do you have previous experience using video consultations? Do you trust the interface? Is it user-friendly and convenient? In what situations can this interface be used? How can the interface be improved to promote confidence and trust in the tool?
4. For those who haven't experienced it: Why haven't you tried it so far? (e.g., technological reasons, always preferring human contact, don't trust the system, the child is more anxious). What are your expectations from video calls?
5. For those who have experienced it: Why did you try it? What led you to do so? Tell us about your experience. Did it meet your expectations?
6. What do you see as the advantages of video consultations?
7. What challenges or disadvantages have you experienced, or do you anticipate experiencing with VC use?
8. Did you/do you expect to manage VC technically? Do you anticipate any problems?
9. Can you tell us how you feel about the service quality and medical care? (sense of time, availability, patience, professional aspects)
   1. Feeling of having more/less time?
   2. How did you feel about pediatrician availability for video calls compared to clinic visits?
   3. How was the pediatrician's attitude in video calls? More/less patient?
   4. Do you feel the pediatrician was able to understand and assess your child's medical condition through video calls? Why or why not?
10. Can you tell us about the child's feelings, self-expression, and sense of professional confidence - How did you feel? How do you think and expect you would feel?
    1. Did you feel more or less comfortable with the pediatrician?
    2. For your child? (feelings during remote conversation - anxious, worried, or relaxed?) Bored?
    3. Were you able or do you believe you'll be able to express yourself and the medical situation as well as in the clinic? In video calls, you can hear intonation, but facial expressions and body language are sometimes less visible.
    4. What is your confidence level regarding medical treatment via video?
11. Due to the war/pandemic, did you try to find alternative solutions to clinic visits? How did the war affect you when medical treatment was needed for your child?

- Closing - Summary and recommendations: Does anyone have anything else to add or comment on?
